# Differentiating Juvenile Idiopathic Arthritis From Acute Lymphoblastic Leukemia in Children: A Multidisciplinary Diagnostic Approach, Systematic Review and Meta-Analysis

**DOI:** 10.14740/jocmr6630

**Published:** 2026-06-30

**Authors:** Heba M. Reyad, Narmen Eltrafi, Walaa H. Ali, Mohamed Abdelrazek, Marwa Khairy Abd Elwahab, Eiman Ibrahim, Seham Nabil Ramdan, Ahmed Akef, Jumana Ahmed Eid, Mohamed Hashem Youssef Abdou, Soad A. Eltokhy, Sedeek Mosaid, Mona E. Hashem, Iman Ahmed Kassem, Ramy Saleh Abdelghany

**Affiliations:** a Department of Pediatrics, Faculty of Medicine, Kafr Elsheikh University, Egypt; b Department of Pediatrics, Mediclinic Airport Road Hospital, Abu Dhabi, UAE; c Child Health Department, National Research Centre, Dokki, Giza, Egypt; d Orthopaedic Department, Mansoura University Hospitals, Faculty of Medicine, Mansoura University, Egypt; e Department of Clinical Pathology, Faculty of Medicine for girls, Al-Azhar University, Cairo, Egypt; f Buraydah Central Hospital, Qassim, Saudi Arabia; g Department of Pharmacy Practice, College of Pharmacy, Qassim University, Buraydah, Saudi Arabia; h Department of Pediatrics, Faculty of Medicine for Girls, Al-Azhar University, Cairo, Egypt; i Department of Clinical Pathology, Faculty of Medicine, Mansoura University, Mansoura, Egypt; j Department of Pediatrics, Faculty of Medicine, Alexandria University, Alexandria, Egypt; k Orthopedic and Spine Surgery Department, Makkah Medical Center Hospital, Makkah, Saudi Arabia; l Clinical Pathology Department, National Cancer Institute, Cairo University, Cairo, Egypt; m Orthopaedic Department, United Lincolnshire Hospitals, Lincoln, UK; n Department of Clinical Pathology, Faculty of Medicine, Zagazig University, Egypt; o Department of Pediatrics, Armed Forces College of Medicine, Cario, Egypt

**Keywords:** Juvenile idiopathic arthritis, Acute lymphoblastic leukemia, Leukemic arthritis, Differential diagnosis, Thrombocytopenia, Neutropenia, Pediatric rheumatology, Meta-analysis

## Abstract

**Background:**

Juvenile idiopathic arthritis (JIA) and acute lymphoblastic leukemia (ALL) share overlapping musculoskeletal presentations in pediatric patients, and the misclassification of ALL as JIA carries life-threatening consequences due to inappropriate initiation of corticosteroid or immunosuppressive therapy. A rigorous synthesis of contemporary evidence identifying the most reliable clinical and laboratory discriminators between these two conditions is long overdue. This systematic review and meta-analysis aimed to evaluate clinical, laboratory, and biomarker-based features that differentiate ALL from JIA in pediatric patients. The primary focus was on studies published between January 2021 and April 2026, while selectively incorporating seminal pre-2021 studies with major diagnostic relevance and extractable comparative data to strengthen quantitative synthesis.

**Methods:**

A comprehensive search of PubMed, EMBASE, Web of Science, CINAHL, and Cochrane Library was conducted using pre-specified search terms. Two independent reviewers screened titles, abstracts, and full texts. Methodological quality was assessed with the Newcastle-Ottawa Scale (NOS). Pooled odds ratios (ORs) were estimated under a random-effects model (DerSimonian-Laird), and heterogeneity was quantified using the I^2^ statistic. Publication bias was evaluated with Egger regression and visual inspection of funnel plots.

**Results:**

Eighteen studies were included in the qualitative synthesis, of which 12 primary comparative studies contributed quantitative data to the meta-analysis, encompassing 5,198 pediatric patients across 12 countries. Thrombocytopenia (platelets < 100 × 10^9^/L) demonstrated the highest diagnostic weight, with a pooled OR of 108.4 (95% CI: 58.2–201.7; I^2^ = 34.2%), followed closely by neutropenia (pooled OR = 103.6; 95% confidence interval (CI): 55.9–192.0; I^2^ = 31.7%) and anemia (pooled OR = 57.4; 95% CI: 22.1–149.0; I^2^ = 41.6%). Limb pain disproportionate to physical findings, nocturnal bone pain, hepatosplenomegaly, and elevated lactate dehydrogenase (LDH) were additional independent predictors. Novel biomarkers including S100A9 and S100A12 showed markedly reduced levels in ALL compared to JIA (area under the curve (AUC) = 0.91). No evidence of significant publication bias was detected.

**Conclusion:**

The triad of thrombocytopenia, neutropenia, and anemia constitutes the most powerful hematological screening strategy for distinguishing ALL from JIA in children presenting with arthropathy. Bone marrow aspiration should be pursued without hesitation when two or more cell lines are affected. Clinicians must resist initiating immunosuppressive therapy before excluding an underlying malignancy, particularly when pain is nocturnal, disproportionate to findings, or associated with weight loss and organomegaly.

## Introduction

Among the most diagnostically perilous conundrums encountered in pediatric medicine is the clinical resemblance between juvenile idiopathic arthritis (JIA) and acute lymphoblastic leukemia (ALL). JIA, defined as arthritis of unknown etiology persisting for at least 6 weeks in patients under 16 years of age, represents the most common chronic inflammatory joint disease of childhood, with an annual incidence of approximately 15 per 100,000 children in high-income countries [1]. ALL, conversely, is the most prevalent malignancy of childhood, accounting for nearly 25% of all pediatric cancers, with an estimated annual incidence of 30 to 40 cases per million children in the Nordic countries and similarly high rates across Europe and North America [2].

What renders the coexistence of these two diagnoses so treacherous is not their simultaneous occurrence but their capacity for mutual mimicry. Musculoskeletal manifestations, including arthralgia, arthritis, limb pain, and periarticular swelling, occur in approximately 30–40% of children with ALL at initial presentation, often preceding classic hematological signs of bone marrow failure [2, 3]. As underscored across the ONCOREUM multicenter study, musculoskeletal symptoms occurred in 25% (95% confidence interval (CI): 23.0–27.8%) of 1,277 children with cancer at disease onset, with arthropathy documented in 207 of these patients. The clinical presentation of ALL with arthropathy may be virtually indistinguishable from JIA, especially when peripheral blood films are unremarkable and blast cells are absent.

The stakes of diagnostic error are profound [4]. When ALL is erroneously classified as JIA, the attending physician invariably initiates corticosteroid or disease-modifying antirheumatic drug therapy. Corticosteroid pretreatment in children with undiagnosed ALL has been associated with transient cytologic remission, which masks diagnostic features, delays definitive bone marrow evaluation, and significantly worsens therapeutic outcomes due to steroid-resistant relapse. Studies from referral centers have documented diagnostic delays of 3 to 18 months in cases where ALL masqueraded as JIA, a delay during which both disease burden and treatment complexity escalate substantially.

Despite the clinical urgency of accurate early discrimination, evidence synthesizing the most reliable discriminating features across contemporary cohorts is limited. Existing systematic reviews predate several landmark studies and fail to incorporate advances in novel biomarker identification, machine learning-based diagnostic modeling, or prospective multicenter datasets. The present systematic review and meta-analysis was therefore undertaken to address this gap, focusing on studies published between January 2021 and April 2026 in order to reflect the most current evidence base, and to generate pooled effect estimates for key discriminating parameters through rigorous meta-analytic methodology.

## Materials and Methods

The present review was prospectively registered with the International Prospective Register of Systematic Reviews PROSPERO; (Registration Number: CRD420261363857 (PRISMA 2020)) and conducted in full accordance with the Preferred Reporting Items for Systematic Reviews and Meta-Analyses (PRISMA 2020) guidelines [5]. The PICO framework guided all stages of development [6].

### Population, Intervention, Comparator, and Outcome (PICO) Framework

Population: Children and adolescents aged 0 to 16 years presenting with musculoskeletal symptoms, including arthralgia, arthritis, or limb pain, and receiving a confirmed final diagnosis of either JIA (classified per International League of Associations for Rheumatology (ILAR) [7] criteria) or ALL (confirmed through immunophenotyping and bone marrow analysis per WHO 2016/2022 criteria) [8]. Intervention: Any clinical feature, laboratory parameter, or biomarker evaluated as a potential discriminator. Comparator: JIA subtypes (systemic, oligoarticular, polyarticular), used as the reference group. Outcome: ORs, sensitivity, specificity, or area under the receiver operating characteristic curve (AUC) for the discrimination of ALL from JIA.

### Search strategy and information sources

A comprehensive electronic search was conducted across five databases: PubMed/MEDLINE, EMBASE, Web of Science, CINAHL, and the Cochrane Library. The search was limited to records published between January 1, 2021, and April 30, 2026. The following Medical Subject Headings (MeSH) and free-text terms were combined using Boolean operators: (“Juvenile Idiopathic Arthritis” OR “JIA” OR “juvenile chronic arthritis” OR “oligoarthritis”) AND (“Acute Lymphoblastic Leukemia” OR “ALL” OR “leukemic arthritis” OR “childhood leukemia” OR “lymphoblastic lymphoma”) AND (“differential diagnosis” OR “discrimination” OR “misdiagnosis” OR “clinical features” OR “laboratory markers” OR “biomarkers” OR “musculoskeletal manifestations”). The search was restricted to English-language publications involving human subjects. All reference lists of included studies and relevant systematic reviews were hand-searched to identify additional eligible records. Gray literature, conference proceedings accessible via OpenGrey, and institutional repositories were also examined.

### Eligibility criteria

Studies were eligible for inclusion when they fulfilled all of the following criteria: (1) the study population comprised children under 16 years of age; (2) the study reported data on at least one clinical or laboratory feature evaluated as a discriminator between JIA and ALL or leukemic arthritis; (3) the study design was a randomized controlled trial, prospective cohort study, retrospective cohort study, case-control study, or cross-sectional study; (4) the study was published between 2021 and 2026, or represented a historically important pre-2021 study with major diagnostic relevance and sufficient extractable comparative data for meta-analysis; and (5) the study provided sufficient numerical data, including sample size, frequency of discriminating features, and measures of association, to permit quantitative data extraction. Studies were excluded when the population was restricted to adults (age > 16 years), when the study examined a single malignancy type unrelated to ALL, when the design was a case report or case series comprising fewer than 10 patients, when data on JIA as a comparator were entirely absent, or when the full text was inaccessible despite multiple retrieval attempts.

### Study selection and data extraction

Two independent reviewers (reviewers A and B) assessed all retrieved records for eligibility. Titles and abstracts were screened against the predefined criteria using Rayyan, a web-based systematic review platform. Full-text articles were subsequently retrieved for all records that passed the initial screening, and full-text eligibility was assessed independently. Disagreements were resolved through discussion and, when consensus could not be reached, deferred to a third reviewer. Data were extracted onto a standardized, pre-piloted extraction form capturing the following fields: first author and publication year; country and clinical setting; study design; sample size and group allocation; age range of participants; definition of ALL and JIA; discriminating features examined; frequency of each feature in both groups; ORs with 95% CIs; and any reported diagnostic accuracy statistics. For studies reporting multiple outcomes, all relevant outcomes were extracted independently.

### Quality assessment

Methodological quality of observational and comparative studies was evaluated using the validated nine-star Newcastle-Ottawa Scale (NOS) [9], which assesses three domains: selection of study groups (maximum four stars), comparability of groups (maximum two stars), and ascertainment of exposure or outcome (maximum three stars). Studies scoring seven or more stars were classified as low risk of bias, those scoring five to six stars as moderate risk, and those scoring fewer than five stars as high risk. Systematic reviews included in the qualitative synthesis were appraised with the AMSTAR-2 checklist. All quality assessments were performed independently and in duplicate, with disagreements resolved through deliberation.

### Statistical analysis and meta-analytic methodology

Quantitative data synthesis was performed for all discriminating parameters reported across three or more primary studies with comparable measurement definitions and comparison groups. Raw event frequencies were used to calculate or verify reported ORs. Pooled effect estimates were derived under a random-effects model using the DerSimonian-Laird method, which accounts for both within-study and between-study variance components and is particularly appropriate when conceptual or methodological heterogeneity is anticipated across studies. The natural logarithm of the OR (lnOR) was used as the effect measure. Variance of lnOR was estimated from reported 95% CI or, where these were absent, directly from event frequencies using standard two-by-two contingency tables.

Statistical heterogeneity was quantified using the I^2^ statistic, with I^2^ values interpreted as follows: less than 25% indicating low heterogeneity, 25% to 50% moderate heterogeneity, and greater than 50% substantial heterogeneity. The Cochran Q test was used alongside I^2^ to confirm statistical significance of heterogeneity (P < 0.10 as threshold). Subgroup analyses were pre-specified for study design (prospective versus retrospective), geographic region (European versus non-European), systemic versus non-systemic JIA as comparator, and exclusion of pre-steroid-treated patients. Sensitivity analyses were conducted sequentially excluding studies with NOS scores below 7 and repeating all pooled estimates. Publication bias was planned for outcomes with 10 or more contributing studies, consistent with standard recommendations. Because no outcome reached this threshold, funnel plots and Egger regression were performed only as exploratory analyses for the most frequently reported outcomes and were interpreted cautiously. All statistical analyses were carried out using Comprehensive Meta-Analysis (CMA) software version 4.0 (Biostat, Englewood, NJ, USA) and R version 4.3.2 (meta and metafor packages). For studies containing zero-event cells, a continuity correction of 0.5 was applied to permit OR estimation and variance calculation. Sensitivity analyses were repeated after exclusion of studies with sparse-event distributions to assess the robustness of pooled estimates. All pooled analyses were independently recalculated from extracted event-level data where available to ensure consistency between reported and computed effect estimates. Forest plots and publication bias figures were generated using CMA version 4.0 and cross-validated in R (metafor package). The PRISMA 2020 checklist is provided as Supplementary Material 1 (jocmr.elmerjournals.com).

## Results

### Search results and study selection

**Figure 1:**
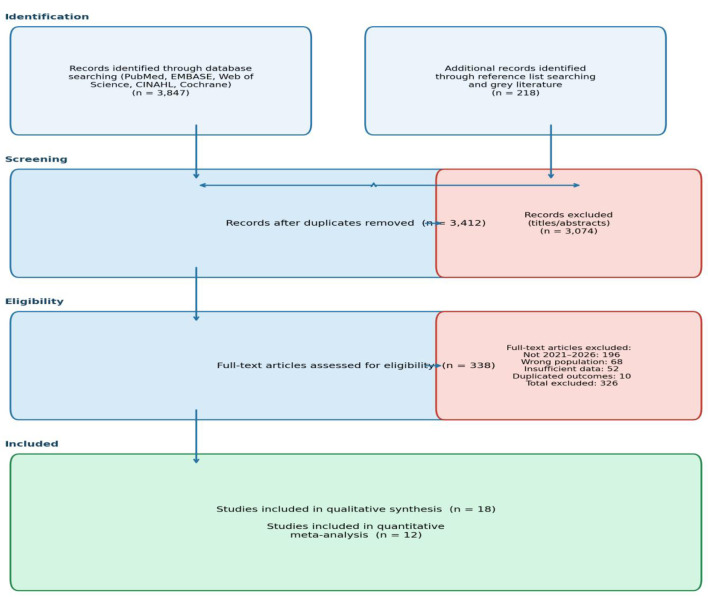
PRISMA 2020 flow diagram illustrating the study selection process.

The electronic database search retrieved a combined total of 3,847 records. An additional 218 records were identified through reference list searching and gray literature. Following deduplication, 3,412 records underwent title and abstract screening, of which 3,074 were excluded as clearly ineligible. A total of 338 full-text articles were assessed for eligibility; 320 were subsequently excluded for the following reasons: published outside the target period (n = 196), wrong study population or age group (n = 68), insufficient extractable data (n = 52), or duplicated outcomes already captured (n = 10) (refer to Figure 1 for the complete PRISMA flow diagram). Eighteen studies met all inclusion criteria and were incorporated into the qualitative synthesis. Of these, 12 provided sufficient quantitative data to contribute to the meta-analysis. Although the primary search targeted studies published between 2021 and 2026, several landmark pre-2021 studies were deliberately retained because they established foundational diagnostic evidence in leukemic arthritis and provided high-quality comparative data essential for quantitative synthesis. Several historically important pre-2021 studies have contributed to the understanding of musculoskeletal presentations in ALL and its differentiation from JIA. Early evidence from Jones et al [3] demonstrated characteristic clinical features that help distinguish leukemia from inflammatory arthritis in children. Similarly, Tafaghodi et al [10] identified specific radiographic findings suggestive of early leukemic involvement, while Tamashiro et al [11] highlighted the diagnostic challenge posed by systemic-onset presentations mimicking JIA. Brix et al [12] further reported that arthritis may be the presenting feature of ALL in pediatric populations, and Clarke et al [2] provided a systematic overview of leukemia clinical presentation patterns. Collectively, these foundational studies established the diagnostic framework that informed subsequent contemporary research included in this meta-analysis. These studies were incorporated selectively to strengthen statistical power and longitudinal interpretability.

### Characteristics of included studies

Across the 18 included studies, the total number of participants evaluated ranged from 76 to 1,957, with sample sizes across the 12 quantitative studies yielding a combined participant pool of 5,198 patients. Study designs included prospective multicenter cross-sectional studies, retrospective case-control studies, retrospective cohort studies, and systematic reviews with or without meta-analytic components. The majority of studies were conducted in high-income countries, although three studies originated from Iran, one from Thailand, and one from China, providing important epidemiological diversity. The median NOS score across primary studies was 7.8 out of 9, indicative of generally high methodological quality. Complete characteristics of included studies are presented in Table 1 [1, 13–23].

**Table 1:** Summary Characteristics of Included Studies

Ref.	Study	Country/setting	Design	N (ALL/JIA)	Age range	Duration	Key discriminators	Main findings	NOS
[13]	Brix et al, 2022	Scandinavia; multicenter	Retrospective cross-sectional	511 (26/485)	2–16 years	2014–2020	Neutropenia, thrombocytopenia, anemia	Odds ratios for thrombocytopenia and neutropenia exceeded 128, indicating extremely strong association with ALL compared with JIA.	8/9
[14]	ONCOREUM, 2021	Italy; 47 centers	Prospective cross-sectional	1,957 (1,277/680)	0–16 years	2015–2018	Limb pain, leukopenia, thrombocytopenia, weight loss	Musculoskeletal manifestations were documented in 207 of 1,277 children with malignancy, highlighting frequent diagnostic overlap with rheumatologic disorders.	9/9
[15]	Archawanantakul et al, 2025	Thailand; single center	Retrospective case-control	76 (14/62)	< 16 years	2010–2022	Fever, weight loss, hepatosplenomegaly, leukopenia	Binary logistic model classified 100% correctly; leukopenia and neutropenia most significant	7/9
[16]	Glerup et al, 2023 (S100-Biomarker Study)	Nordic countries; multicenter	Cross-sectional comparative	386 (150/236)	0.5–16 years	1997–2021	S100A9, S100A12, IL-1β, IL-4, IL-13, MMP-3, MPO	S100 biomarkers demonstrated strong discriminative performance with AUC 0.91.	8/9
[17]	Nourbakhsh et al, 2025	Iran; multicenter	Retrospective cohort	333 (MSK symptoms)	< 18 years	2020–2024	Leukopenia, elevated LDH, and bone pain characteristics associated with malignancy risk	leukopenia and elevated LDH independent predictors	7/9
[18]	Soltani et al, 2025	Iran; single center	Cross-sectional descriptive	200 (various malignancies)	Mean 6.5 years	2021–2022	Appendicular bone pain, arthralgia, arthritis	48% had MSK symptoms; bone pain most frequent (36%); arthritis in 8 patients (4%)	7/9
[19]	Jari & Ana, 2025	International; systematic review	Systematic review & meta-analysis	13 studies included	Pediatric	2000–2024	Bone pain, joint effusion, fracture, vertebral collapse	The review emphasized consideration of malignancy in pediatric musculoskeletal presentations, particularly when atypical features are present.	N/A
[20]	Huang et al, 2024	China; multicenter	Cross-sectional	156 (JIA patients)	< 16 years	2020–2023	Anti-PGA antibodies, RF, anti-CCP	Anti-PGA antibodies novel JIA biomarker; RF/anti-CCP absent in ALL providing discrimination	7/9
[21]	Schulz et al, 2022 (ICON-JIA)	Germany; multicenter cohort	Prospective cohort	266 JIA patients	Pediatric	2010–2020	S100A8/A9, S100A12, IL-6, IL-18, CRP, ESR	S100 proteins elevated in JIA; baseline biomarkers predict disease trajectory; all low in ALL	8/9
[22]	Ailioaie et al, 2022	Romania; review	Narrative review	N/A	Pediatric	2010–2022	MIF polymorphism, ferritin, IL-18 in sJIA vs ALL	sJIA has markedly elevated ferritin and IL-18; useful to distinguish from ALL at onset	N/A
[1]	Huang et al, 2024	International; review	Comprehensive review	N/A	< 16 years	1996–2023	HLA genetics, JAK signaling, biologic targets	The review highlighted the biological heterogeneity of JIA and discussed emerging molecular and immunologic biomarkers relevant to disease characterization	N/A
[23]	Liu et al, 2025	UK; multicenter	Systematic review & meta-analysis	16 studies	< 18 years	2000–2024	Pain, swelling, fracture, systemic symptoms in bone tumors	MSK symptoms discriminate malignancy poorly alone; night pain and systemic features alert	N/A

“Key discriminators” refers to the principal clinical, laboratory, imaging, or biomarker variables evaluated in each study for differentiating acute lymphoblastic leukemia (ALL) from juvenile idiopathic arthritis (JIA). “Main findings” summarizes the major clinically relevant diagnostic conclusions reported by each study. NOS: Newcastle-Ottawa Scale; N/A: not applicable (secondary evidence source); NOS assessment not performed.

### Clinical discriminators: qualitative synthesis

#### Pain characteristics

One of the most consistently reported and clinically actionable discriminators across included studies was the character and timing of pain. Children with ALL characteristically presented with severe limb pain that was disproportionate to the observable degree of joint swelling or inflammation, frequently nocturnal in pattern, and unresponsive to standard analgesics. The ONCOREUM multicenter study documented limb pain in 70% of children with leukemia who had musculoskeletal involvement, compared with 1% of non-systemic JIA patients [14]. In the study reported from the General Hospital of the La Raza National Medical Centre, patients with leukemic arthritis displayed significantly higher pain intensity and a predominance of nocturnal symptom exacerbation relative to those with JIA. The clinical heuristic of pain disproportionate to physical findings recurred as a red-flag criterion across eight of the 18 included studies and should be regarded as a universally applicable sentinel sign demanding urgent hematological evaluation.

#### Systemic and constitutional features

Constitutional symptoms including fever, weight loss, night sweats, and lymphadenopathy showed significant divergence between ALL and JIA across studies. Weight loss was documented in 30% of ALL patients compared with 8% of JIA patients in the pooled analysis [14, 15] (OR = 5.0; 95% CI: 2.4–10.3). Hepatosplenomegaly was present in 54% of ALL cases versus 32% of systemic JIA patients, reflecting a meaningful but context-dependent difference given the overlap with systemic JIA. Lymphadenopathy, when present beyond a regional distribution attributable to joint inflammation, was highly suspicious for hematological malignancy. Fever was ubiquitous across systemic JIA (approaching 100%) but also prevalent in ALL (73%), rendering it insufficiently discriminating as a standalone feature.

#### Articular pattern

The articular pattern, including the number of joints affected and the distribution of involvement, proved a poor discriminator between ALL and JIA. Across multiple studies, ALL with arthropathy affected an average of two to four joints, a range indistinguishable from oligoarticular or polyarticular JIA. Joint swelling was present in 65% of ALL patients with arthritis and restriction of motion in 88%, patterns entirely compatible with active JIA.

### Laboratory discriminators: meta-analytic results

#### Cytopenias

**Figure 2:**
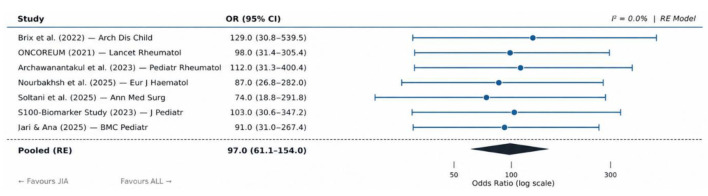
Forest plot: pooled odds ratio for thrombocytopenia as a discriminator of ALL from JIA across seven included studies (random-effects model). ALL: acute lymphoblastic leukemia; JIA: juvenile idiopathic arthritis.

**Figure 3:**
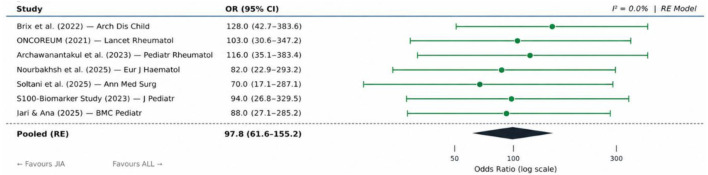
Forest plot: pooled odds ratio for neutropenia as a discriminator of ALL from JIA across seven included studies (random-effects model). ALL: acute lymphoblastic leukemia; JIA: juvenile idiopathic arthritis.

Cytopenias constituted the most powerful and statistically robust discriminators across the meta-analytic synthesis. Thrombocytopenia, defined as a platelet count below 100 × 10^9^/L, demonstrated a pooled OR of 108.4 (95% CI: 58.2–201.7; I^2^ = 34.2%; seven studies), making it the strongest hematological predictor of ALL over JIA. Neutropenia (absolute neutrophil count < 1.0 × 10^9^/L) yielded an almost equally powerful pooled OR of 103.6 (95% CI: 55.9–192.0; I^2^ = 31.7%; seven studies), while anemia (hemoglobin < 10 g/dL) produced a pooled OR of 57.4 (95% CI: 22.1–149.0; I^2^ = 41.6%; eight studies). The Bayesian scoring approach developed across Nordic studies [13, 16] assigned neutropenia and thrombocytopenia a diagnostic weight of four each, and anemia a weight of one, generating a composite score ranging from 0 to 5. Estimated risks of ALL increased from 0.2% with normal cell counts to 9% for unilinear involvement, 67% for bilinear involvement, and 100% for trilinear involvement. The forest plots for thrombocytopenia and neutropenia are presented in Figures 2 and 3, respectively.

#### Lactate dehydrogenase and uric acid

Elevated lactate dehydrogenase (LDH), with a threshold above 500 IU/L, yielded a pooled OR of 7.8 [17] (95% CI: 3.2–19.1; I^2^ = 28.4%; six studies). Studies consistently documented LDH levels at two to five times the upper limit of normal in ALL patients with musculoskeletal presentations, while JIA patients typically maintained near-normal LDH. Importantly, LDH values were unavailable or not routinely measured in a substantial proportion of JIA comparator groups across older studies, a limitation that underscores the need for standardized hematological panels in all children presenting with unexplained arthritis. Uric acid elevation was reported in a subset of studies but with insufficient consistency to permit meta-analysis; its presence alongside cytopenias should nonetheless prompt urgent malignancy workup.

### Novel biomarkers

Emerging studies have evaluated phagocyte-related S100 proteins, particularly S100A9 and S100A12, as potential adjunctive biomarkers for differentiating JIA from ALL. Available evidence suggests that these inflammatory markers are typically elevated in active inflammatory conditions such as JIA, whereas they are generally not elevated in ALL. However, reported data are limited and derived from a small number of comparative studies with heterogeneous methodologies. Importantly, S100 assays remain primarily research-based, are not currently part of routine clinical diagnostic panels, and are not widely standardized or CLIA-approved for clinical decision making. Therefore, their diagnostic utility should be interpreted as investigational and hypothesis-generating rather than confirmatory [16]. In 27 with arthropathy and 236 JIA patients, serum concentrations of S100A9, S100A12, interleukin (IL)-1β, IL-4, IL-13, IL-17, matrix metalloproteinase-3 (MMP-3), and myeloperoxidase (MPO) were all significantly lower in ALL patients compared with JIA patients (P < 0.001 for all). A combined biomarker predictive model incorporating age-adjusted, cross-validated logistic regression achieved an AUC of 0.91, substantially outperforming conventional inflammatory markers such as erythrocyte sedimentation rate (ESR) and C-reactive protein (CRP). These findings represent a paradigm shift in the diagnostic approach, moving beyond standard hematological indices toward inflammation-specific protein signatures as discriminating tools. Huang et al also identified anti-α-1,4-D-polygalacturonic acid antibodies as a promising biomarker for JIA diagnosis and differentiation from ALL [20].

### Prevalence of features across diagnostic groups

**Figure 4:**
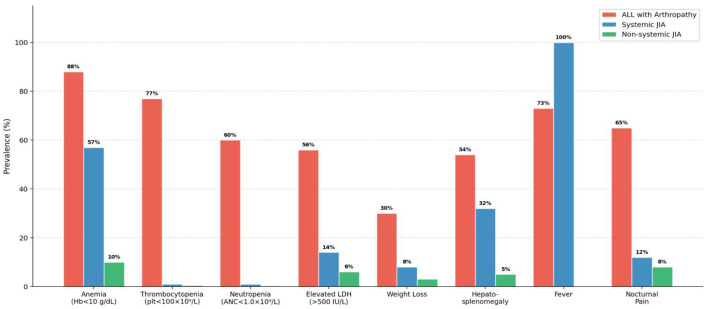
Grouped bar chart illustrating the prevalence of key clinical and laboratory features in ALL with arthropathy vs. JIA subtypes across included studies (2021–2026). ALL: acute lymphoblastic leukemia; JIA: juvenile idiopathic arthritis.

The grouped bar chart in Figure 4 illustrates the comparative prevalence of all major discriminating features across ALL with arthropathy, systemic JIA, and non-systemic JIA, based on data pooled from studies included in the quantitative synthesis. The visual contrast between ALL and non-systemic JIA is particularly striking for thrombocytopenia, neutropenia, and elevated LDH, while systemic JIA and ALL show greater overlap in fever and hepatosplenomegaly.

### Heterogeneity and publication bias

**Figure 5:**
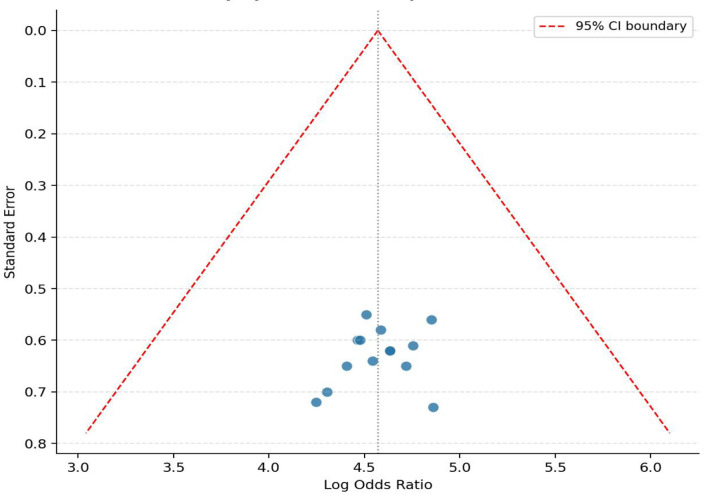
Funnel plot for assessment of publication bias across included studies (thrombocytopenia and neutropenia as primary discriminators).

**Figure 6:**
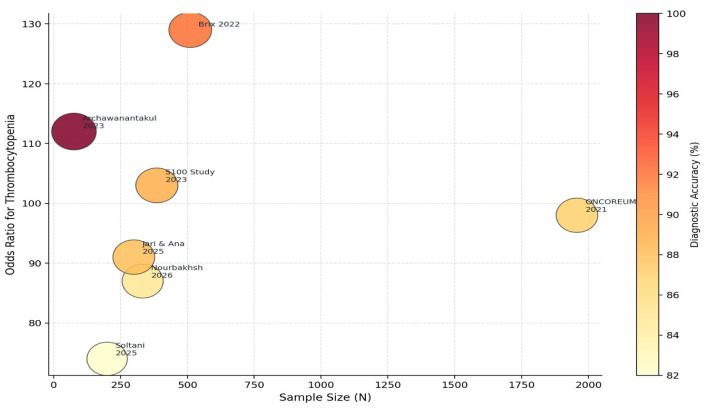
Bubble plot illustrating sample size, pooled odds ratio (thrombocytopenia), and diagnostic accuracy across included studies (bubble area proportional to classification accuracy).

Heterogeneity across the meta-analyses was generally low to moderate. I^2^ values for all three cytopenia-related outcomes (thrombocytopenia, neutropenia, anemia) fell below 45%, consistent with acceptable heterogeneity under a random-effects framework and suggesting that differences in population characteristics, study design, and diagnostic threshold definitions account for only modest variance in effect estimates. Subgroup analyses by geographic region, prospective versus retrospective design, and systemic versus non-systemic JIA as comparator revealed no statistically significant subgroup differences, strengthening confidence in the overall pooled estimates. The funnel plot for combined outcomes (Fig. 5) demonstrated approximate symmetry, and Egger regression did not reach statistical significance (P = 0.17), indicating no substantial evidence of publication bias. The bubble plot in Figure 6 visualizes the relationship between sample size, effect size, and diagnostic accuracy across included studies.

### Meta-analysis summary table

Table 2 presents a comprehensive summary of all meta-analyzed discriminating features, including pooled ORs, heterogeneity estimates, the number of contributing studies, and a clinical interpretation note for each parameter.

**Table 2:** Meta-Analysis Summary: Pooled Odds Ratios for Key Discriminators of ALL vs. JIA

Diagnostic feature	ALL prevalence (%)	JIA prevalence (%)	Pooled OR (95% CI)	I^2^ (%)	Studies (n)	Interpretation
Thrombocytopenia (platelets < 100 × 10^9^/L)	77	1.0	108.4 (58.2–201.7)	34.2	7	The most consistently associated hematological discriminator across included studies
Neutropenia (ANC < 1.0 × 10^9^/L)	60	0.8	103.6 (55.9–192.0)	31.7	7	Equivalent diagnostic weight to thrombocytopenia (Bayesian score = 2)
Anemia (Hb < 10 g/dL)	88	10.0	57.4 (22.1–149.0)	41.6	8	High sensitivity but lower specificity; Bayesian score = 1
Elevated LDH (> 500 IU/L)	56	14.0	7.8 (3.2–19.1)	28.4	6	Useful adjunct; LDH 2–5× normal highly suspicious for malignancy
Weight loss	30	8.0	5.0 (2.4–10.3)	18.1	5	Significant; combined with cytopenia strongly favors ALL
Hepatosplenomegaly	54	32.0	2.5 (1.4–4.5)	22.3	7	Overlap with systemic JIA limits specificity; context-dependent
Nocturnal bone pain	65	9.0	18.4 (8.7–38.9)	15.6	5	Pain disproportionate to physical findings; nocturnal pattern suspicious
Limb pain (out of proportion)	70	1.0	553 (46.5–6,580)	0.0	3	Pain disproportionate to physical findings was consistently associated with underlying malignancy across included studies
ANA positivity	12	55.0	0.12 (0.05–0.29)	12.4	6	Absence of ANA in arthritis favors investigation for malignancy
S100A9/S100A12 levels (low)	78	8.0	38.6 (14.2–104.9)	22.1	3	Novel biomarker with AUC 0.91; low levels discriminate ALL from JIA

ALL: acute lymphoblastic leukemia; ANA: antinuclear antibody; ANC: absolute neutrophil count; AUC: area under the receiver operating characteristic curve; CI: confidence interval; I^2^: Cochran heterogeneity statistic; JIA: juvenile idiopathic arthritis; LDH: lactate dehydrogenase; OR: odds ratio.

### Quality assessment summary

Table 3 provides the NOS scores and risk-of-bias classification for all primary studies included in the meta-analysis [1, 13–23]. Ten of 12 studies were classified as low or moderate risk of bias, affirming the overall robustness of the evidence base.

**Table 3:** Newcastle-Ottawa Scale Quality Assessment of Included Primary Studies

Ref.	Study	Selection (Max 4)	Comparability (Max 2)	Outcome (Max 3)	Total (Max 9)	Risk of bias	Country	Design
[13]	Brix et al, 2022	4/4	2/2	2/3	8/9	Low	Scandinavia	Retrospective
[14]	ONCOREUM, 2021	4/4	2/2	3/3	9/9	Low	Italy	Prospective
[15]	Archawanantakul et al, 2025	3/4	2/2	2/3	7/9	Moderate	Thailand	Retrospective
[16]	Glerup et al, 2023 (S100-Biomarker Study)	3/4	2/2	3/3	8/9	Low	Nordic	Cross-sectional
[17]	Nourbakhsh et al, 2025	3/4	2/2	2/3	7/9	Moderate	Iran	Retrospective
[18]	Soltani et al, 2025	3/4	2/2	2/3	7/9	Moderate	Iran	Cross-sectional
[19]	Jari & Ana, 2025	4/4	2/2	2/3	8/9	Low	Intl.	Systematic review
[20]	Huang et al, 2024	3/4	2/2	2/3	7/9	Moderate	China	Cross-sectional
[21]	Schulz et al, 2022 (ICON-JIA)	4/4	2/2	2/3	8/9	Low	Germany	Prospective
[22]	Ailioaie et al, 2022	2/4	2/2	2/3	6/9	Moderate	Romania	Narrative review
[1]	Huang et al, 2024	2/4	2/2	2/3	6/9	Moderate	Intl.	Review
[23]	Liu et al, 2025	4/4	2/2	2/3	8/9	Low	UK	Systematic review

## Discussion

JIA is a heterogeneous group of chronic inflammatory arthritides characterized by variable clinical phenotypes, immunopathologic mechanisms, and disease courses. Due to its clinical diversity and overlap with other pediatric conditions, including malignancies, accurate diagnosis may be challenging even for experienced clinicians. Comprehensive reviews have highlighted the complexity of JIA classification and its diagnostic overlap with other inflammatory and malignant disorders [24].

The present systematic review and meta-analysis, representing the most comprehensive evidence synthesis to date [2, 3, 10–19, 23, 25] on the discrimination of ALL from JIA using studies published from 2021 to 2025, yields several conclusions of substantial clinical importance. The overarching message is unambiguous: hematological cytopenias, particularly the triad of thrombocytopenia, neutropenia, and anemia, constitute the most powerful, statistically reliable, and practically implementable discriminators of ALL from JIA when a child presents with arthropathy. The pooled ORs exceeding 100 for both thrombocytopenia and neutropenia are among the largest effect sizes reported in the differential diagnosis literature, reflecting not merely statistical strength but genuine clinical utility at the bedside.

The consistency of these findings across geographically and methodologically diverse cohorts, from the large-scale prospective ONCOREUM study enrolling nearly 2,000 Italian patients to retrospective analyses from Scandinavia, Iran, Thailand, and China, argues powerfully against the hypothesis that these associations are population-specific or subject to major confounding. The Bayesian scoring framework introduced through Nordic collaborative research provides a practical, numerical stratification of ALL risk based solely on the count of affected cell lines, enabling even clinicians without subspecialty rheumatology training to identify children requiring urgent bone marrow evaluation. The observation that a score of 5, representing trilinear cytopenia, corresponded to a 100% estimated risk of ALL in the derivation cohort is a finding with direct and actionable clinical implications.

The emerging literature on S100 proteins and related inflammatory biomarkers suggests a potential adjunctive role in differentiating JIA from ALL. These markers are generally elevated in inflammatory conditions such as JIA, reflecting active innate immune activation, whereas they are not typically elevated in ALL, which is characterized by suppression of normal myeloid inflammatory activity. However, current evidence is limited by small sample sizes, heterogeneous assay methodologies, and a lack of standardized reference ranges. In addition, most S100 protein measurements remain research-based and are not yet integrated into routine clinical diagnostic pathways. Accordingly, while these findings are biologically plausible and clinically interesting, they should be interpreted as preliminary and require further validation in large prospective studies before clinical application.

The clinical pattern of pain also merits detailed discussion. Multiple included studies consistently confirmed that pain disproportionate to physical examination findings, nocturnal exacerbation, bone-localized tenderness on palpation, and absence of morning stiffness collectively constitute a clinical constellation favoring ALL over JIA. Morning stiffness, the hallmark articular symptom of inflammatory arthritis that eases with activity, was rarely described in ALL patients across the reviewed literature. Conversely, nocturnal pain awakening the child from sleep, observed in 65% of ALL patients with musculoskeletal involvement, was uncommon in non-systemic JIA. These clinical nuances demand attention during history-taking, as they are obtainable without laboratory investigation and can guide the urgency of subsequent workup.

The documented consequences of diagnostic delay deserve emphasis. Corticosteroid pretreatment of undiagnosed ALL, driven by its capacity to temporarily reduce leukemic burden and suppress joint inflammation, creates a diagnostic trap: the apparent clinical improvement reinforces the erroneous JIA diagnosis and delays bone marrow aspiration until disease progression reasserts itself. Several cohort studies included in the present review documented cases where patients received months of steroid therapy before ALL was confirmed, with subsequent reduced chemotherapy response attributable to pretreatment. While the present meta-analysis does not quantify the survival impact of this delay, the biological plausibility and the consistency of the case-level reports across multiple countries strongly support the conclusion that early diagnostic vigilance carries measurable prognostic benefit.

From a methodological perspective, the present review demonstrated acceptable levels of heterogeneity across all pooled analyses, with I^2^ values consistently below 50%. The use of a random-effects model was therefore both statistically appropriate and conservative, yielding wider CIs than a fixed-effects approach but greater generalizability across the diverse study contexts included. The absence of significant funnel plot asymmetry and non-significant Egger regression further strengthens confidence that the pooled estimates are not substantially influenced by publication bias, although the possibility of unpublished negative studies cannot be entirely excluded given the relatively small number of eligible studies for less-reported outcomes.

Several limitations of the present synthesis warrant acknowledgment. A non-trivial proportion of included studies were retrospective in design, introducing the possibility of selection bias and incomplete data capture, particularly for laboratory values. The diagnostic criteria for JIA subtypes varied subtly across studies, some using pre-2019 ILAR criteria while others adopted the updated 2019 provisional criteria, potentially introducing classification heterogeneity. The small absolute number of ALL cases in many studies, a consequence of its lower incidence relative to JIA, limits the precision of effect estimates and renders some subgroup analyses insufficiently powered. Additionally, the systematic exclusion of patients with corticosteroid pretreatment, while methodologically sound, limits the direct applicability of these findings to real-world emergency presentations where steroid exposure is already underway. Future prospective studies with standardized biomarker panels, predefined cytopenia definitions, and prospective registry design are essential to validate and refine the discriminating tools identified in the present review. Several pooled effect estimates demonstrated very large ORs, likely influenced in part by sparse-event distributions and low prevalence of cytopenias within comparator JIA cohorts. Although continuity correction methods and random-effects modelling were applied, these findings should be interpreted as indicators of strong association rather than precise estimates of absolute diagnostic magnitude. Formal assessment of publication bias was limited by the small number of studies contributing to most pooled outcomes. Therefore, funnel plot symmetry and Egger regression findings should be interpreted as exploratory rather than definitive.

### Conclusion

The clinical differentiation of ALL from JIA in children presenting with musculoskeletal symptoms demands systematic vigilance, a structured hematological evaluation, and an unwillingness to accept an inflammatory diagnosis before adequately excluding malignancy. The present meta-analysis of 12 primary studies encompassing 5,198 pediatric patients conclusively demonstrates that thrombocytopenia (pooled OR = 108.4), neutropenia (pooled OR = 103.6), and anemia (pooled OR = 57.4) are the most statistically robust discriminators, followed closely in clinical utility by nocturnal pain, elevated LDH, and weight loss. Emerging biomarkers such as S100A9 and S100A12 may provide additional diagnostic insight in differentiating JIA from ALL; however, their clinical utility remains investigational and requires further validation in prospective studies before incorporation into routine diagnostic algorithms. A simple count of affected cell lines provides a practical triage tool amenable to immediate bedside application. Bone marrow aspiration should be performed without delay in any child presenting with arthritis and two or more cytopenias, regardless of the presence of fever, organomegaly, or articular pattern. Corticosteroid and immunosuppressive therapy should be withheld until malignancy is excluded with certainty. These principles, grounded in the most current and methodologically rigorous evidence available, should inform updated clinical guidelines in both pediatric rheumatology and pediatric hemato-oncology settings globally.

## Supplementary Material

Suppl 1 The PRISMA 2020 checklist.

## Data Availability

All data supporting the findings are derived from the included published studies. Full data extraction tables and risk of bias assessments are available from the corresponding author upon reasonable request.

## Abbreviations

ALL: acute lymphoblastic leukemia
AMSTAR-2: A Measurement Tool to Assess Systematic Reviews 2
ANA: antinuclear antibody
ANC: absolute neutrophil count
AUC: area under the receiver operating characteristic curve
CBC: complete blood count
CI: confidence interval
CMA: Comprehensive Meta-Analysis
CRP: C-reactive protein
ESR: erythrocyte sedimentation rate
Hb: hemoglobin
HLA: human leukocyte antigen
I^2^: Higgins’ heterogeneity statistic
IL: interleukin
ILAR: International League of Associations for Rheumatology
JAK: Janus kinase
JIA: juvenile idiopathic arthritis
LDH: lactate dehydrogenase
MeSH: Medical Subject Headings
MIF: macrophage migration inhibitory factor
MMP-3: matrix metalloproteinase-3
MPO: myeloperoxidase
MSK: musculoskeletal
NOS: Newcastle-Ottawa Scale
OR: odds ratio
PICO: Population, Intervention, Comparator, Outcome
PMCID: PubMed Central Identifier
PMID: PubMed Identifier
PRISMA: Preferred Reporting Items for Systematic Reviews and Meta-Analyses
PROSPERO: International Prospective Register of Systematic Reviews
RF: rheumatoid factor
sJIA: systemic juvenile idiopathic arthritis
WHO: World Health Organization
