# Supplementary material for: Differentiating Juvenile Idiopathic Arthritis From Acute Lymphoblastic Leukemia in Children: A Multidisciplinary Diagnostic Approach, Systematic Review and Meta-Analysis
Source: J Clin Med Res. 2026 Jun 30;18(6):394–406. doi: 10.14740/jocmr6630 (PMC13375429; doi:10.14740/jocmr6630)
Supplement: Suppl 1 — The PRISMA 2020 checklist. [file jocmr-18-06-394-s001.docx]

| **Section and Topic** | **Item No.** | **Checklist Item** | **Reported on Page No.** |
| --- | --- | --- | --- |
| TITLE | 1 | Identify the report as a systematic review and/or meta-analysis | 1 |
| ABSTRACT | 2 | See PRISMA 2020 for Abstracts checklist | 1–2 |
| INTRODUCTION |  |  |  |
| Rationale | 3 | Describe the rationale for the review in the context of existing knowledge | 2–3 |
| Objectives | 4 | Provide an explicit statement of the objectives/questions addressed | 1–3 |
| METHODS |  |  |  |
| Eligibility criteria | 5 | Specify inclusion and exclusion criteria and grouping methods | 4 |
| Information sources | 6 | Specify all databases, registers, websites, organizations, and other sources searched | 4 |
| Search strategy | 7 | Present full search strategies including filters and limits used | 4 |
| Selection process | 8 | Specify methods used to decide study inclusion | 4 |
| Data collection process | 9 | Specify methods used for data extraction | 4–5 |
| Data items | 10a | List and define all outcomes sought | 4–5 |
| Data items | 10b | List and define other variables sought | 4–5 |
| Study risk of bias assessment | 11 | Specify methods used to assess risk of bias | 5 |
| Effect measures | 12 | Specify effect measures used for each outcome | 5 |
| Synthesis methods | 13a | Describe process used to decide study eligibility for synthesis | 5 |
| Synthesis methods | 13b | Describe methods required to prepare data | 5 |
| Synthesis methods | 13c | Describe methods used to tabulate or visually display results | 5–6 |
| Synthesis methods | 13d | Describe synthesis methods and rationale | 5–6 |
| Synthesis methods | 13e | Describe methods to explore heterogeneity | 5–6 |
| Synthesis methods | 13f | Describe sensitivity analyses conducted | 5–6 |
| Reporting bias assessment | 14 | Describe methods used to assess risk of bias due to missing results | 5–6 |
| RESULTS |  |  |  |
| Study selection | 15 | Describe search and selection results from screening to inclusion | 6 |
| Study selection | 16 | Cite studies excluded despite appearing eligible | 6 |
| Study characteristics | 17 | Cite each included study and present characteristics | 7–10 |
| Risk of bias in studies | 18 | Present risk of bias assessments | 16 |
| Results of individual studies | 19 | Present summary statistics and effect estimates | 10–16 |
| Results of syntheses | 20a | Summarize contributing study characteristics and bias | 10–18 |
| Results of syntheses | 20b | Present statistical syntheses results | 11–16 |
| Results of syntheses | 20c | Present heterogeneity investigations | 13 |
| Results of syntheses | 20d | Present sensitivity analyses | 5–6, 13 |
| Reporting biases | 21 | Present assessments of reporting bias | 13–14 |
| DISCUSSION |  |  |  |
| Discussion | 22 | Provide general interpretation of results | 17–18 |
| Discussion | 23a | Discuss limitations of included evidence | 18 |
| Discussion | 23b | Discuss limitations of review processes | 18 |
| Discussion | 23c | Discuss implications for practice and future research | 17–19 |
| OTHER INFORMATION |  |  |  |
| Registration and protocol | 24a | Provide registration information | 3 |
| Registration and protocol | 24b | Indicate where protocol can be accessed | 3 |
| Registration and protocol | 24c | Describe amendments to protocol | Not applicable |
| Support | 25 | Describe financial or non-financial support | 19 |
| Competing interests | 26 | Declare competing interests | 19 |
| Availability of data | 27 | Report availability of data/materials/code | 19 |
